# Systematic Review of Guidelines on Antihypertensive Treatment in Older Adults: SPRINTing to More Heterogeneity?

**DOI:** 10.1093/geroni/igaa057.686

**Published:** 2020-12-16

**Authors:** Jonathan Bogaerts, Leonie von Ballmoos, Wilco Achterberg, Jacobijn Gussekloo, Sven Streit, Milly van der Ploeg, Yvonne Drewes, Rosalinde Poortvliet

**Affiliations:** 1 Leiden University Medical Center, Leiden, Zuid-Holland, Netherlands; 2 University of Bern, Bern, Bern, Switzerland; 3 Leiden University, Leiden, The Hague, Netherlands

## Abstract

Clinical trials have demonstrated that antihypertensive treatment (AHT) in older adults is beneficial. Longitudinal studies, in contrast, have shown that low blood pressure is associated with higher all-cause mortality, especially in frail older adults. Despite the high quality of the available evidence, its translation into clinical guidance for the heterogeneous older population is challenging. To give a systematic overview of blood pressure targets for older adults recommended in clinical guidelines, we searched PubMed, Embase, Emcare, and five guideline databases. We selected guidelines with numerical thresholds for the initiation or the goal of non-disease-specific AHT (January 2008-October 2019). Guidelines with advices concerning AHT in older adults were analyzed. We appraised the guideline quality with the AGREEII-instrument. Of the 44 guidelines containing a numerical threshold for the initiation or the goal of AHT, 33 (75%) provided recommendations concerning AHT for older adults. Nineteen advised a higher target of systolic blood pressure (SBP) for older adults in comparison with the middle-aged population and 3 more recent advised a lower target. Over half (19/33) recommended to treat hypertension in the oldest old to a SBP <150 mmHg, while others advised intensive treatment to targets <120 mmHg. Although many guidelines mentioned frailty, only three gave specific thresholds and targets for frail older adults. The quality of the guidelines was not related with the recommended targets. Targets of AHT in older adults in international guidelines range from less strict to more intensive in comparison with the middle-aged and are set on chronological rather than biological age.

